# Malocclusion-Related Quality of Life Questionnaire (MRQoLQ): Development and validation of a new psychometric tool for older adolescents with malocclusion

**DOI:** 10.1590/2177-6709.24.6.028-035.oar

**Published:** 2019

**Authors:** Elbe Peter, Radha Madhavanpillai Baiju, Remadevi Shivaraman, Netiyatt Ommen Varghese, Jolly Mary Varughese

**Affiliations:** 1 Government Dental College Kottayam, Department of Orthodontics (Kottayam, India).; 2 Government Dental College Kottayam, Department of Periodontics (Kottayam, India).; 3 Kerala University of Health Sciences, School of Health Policy and Planning Studies (Trivandrum, India).; 4 PMS Dental College, Department of Endodontics (Trivandrum, India).; 5 Government of Kerala, Directorate of Medical Education (Trivandrum, India).

**Keywords:** Malocclusion-Related Quality of Life, OHRQoL, Psychometric tool, PROMs in Orthodontics

## Abstract

**Objective:**

To develop and validate a new psychometric tool for assessing malocclusion-related quality of life among older Indian adolescents.

**Methods:**

Item generation involved analysis of existing validated tools, followed by development of new items using various qualitative steps. A draft item pool of 41 questions was initially generated and subjected to item reduction through sequential steps involving two clinical studies to ensure reliability and validity. 431 subjects aged between 15 to 18 years took part in the validation study. Principal component analysis with varimax rotation was performed to get a psychometric tool with good factorial structure and maximum variance.

**Results:**

Rotated component matrix resulted in a 20 item psychometric tool containing 4 domains with a total variance of 61.57%. Inter item, item total correlation and Cronbach α (α = 0.88) ensured good reliability. A positive correlation of the scale with global question ensured convergent validity. Independent *t* test showed statistically significant difference (*p*< 0.05) between mean score of IOTN-DHC and DAI with MRQoLQ, ensuring good construct validity.

**Conclusions:**

The newly developed psychometric tool is named as Malocclusion-Related Quality of Life Questionnaire (MRQoLQ) having 20 questions, including 2 socioeconomic items. The scale showed good reliability and initial validity, hence can be used among older adolescents with malocclusion to assess their malocclusion-related quality of life.

## RESUMO

**Objetivo:**

desenvolver e validar uma nova ferramenta psicométrica para avaliar o impacto da má oclusão na qualidade de vida em pacientes indianos ao final da adolescência.

**Método:**

o delineamento do conjunto de itens desse questionário envolveu a análise de ferramentas existentes já validadas, seguida da criação de novos itens, usando vários passos qualitativos. Um conjunto provisório de itens, com 41 perguntas, foi inicialmente formulado e submetido à redução de itens por meio de passos sequenciais envolvendo dois estudos clínicos, para garantir sua validade e confiabilidade. Nesse estudo de validação, participaram 431 pacientes com idades entre 15 e 18 anos. Foi realizada uma análise do componente principal pelo método Varimax, para se obter uma ferramenta psicométrica com boa estrutura fatorial e variância máxima.

**Resultados:**

a matriz, após as rotações dos fatores, resultou em uma ferramenta psicométrica com 20 itens, contendo 4 domínios, com uma variância total de 61,57%. As correlações interitens e item-total e o alfa de Cronbach (α = 0,88) confirmaram uma boa confiabilidade. Uma correlação positiva da escala com a questão global assegurou validade convergente. O teste *t* independente demonstrou diferença estatisticamente significativa (*p*< 0,05) entre a pontuação média do IOTN-DHC e do DAI com o MRQoLQ, assegurando uma validade bem construída.

**Conclusões:**

essa nova ferramenta psicométrica desenvolvida, denominada Questionário “Impacto da Má Oclusão na Qualidade de Vida” (MRQoLQ, do inglês *Malocclusion-Related Quality of Life Questionnaire*), com 20 perguntas, incluindo 2 itens socioeconômicos, demonstrou boa confiabilidade e validade inicial. Assim, pode ser utilizado em pacientes no final da adolescência, para avaliar o impacto da má oclusão em sua qualidade de vida.

## INTRODUCTION

Malocclusion is due to anatomic variations, and orthodontic treatment aims to correct these variations from an arbitrary norm.^1^ Traditionally malocclusion and its correction by orthodontic treatment is assessed using normative means, ignoring the psychological impacts or benefits.^2^ ^,^ ^3^ There has been a paradigm shift in this concept with the introduction of psychometric scales to measure this intangible dimension of health.^4^ ^-^ ^10^

Children with malocclusions are often subjected to bullying in schools.^11^ They, even at the age of 8 or 9 are conscious about their appearance. The impact of malocclusion on quality of life may be different in children than in adults, due to difference in psychological, social and emotional factors.^5^ Literature search revealed many generic and condition-specific tools for orthodontic use.^4^ ^,^ ^5^ ^,^ ^7^ ^-^ ^10^ ^,^ ^12^ Marshman and Robinson^13^ have expressed the inappropriateness of using generic questionnaires for subjects with malocclusion. Among the available condition-specific tools,^7-10^ Orthognathic Quality of Life Questionnaire (OQLQ) is exclusively for orthognathic patients^7^ ^,^ ^8^ and Psychosocial Impact of Dental Aesthetics Questionnaire (PIDAQ) is primarily for adults.^9^ The recently published Malocclusion Impact Questionnaire (MIQ) developed in UK can be used for young adolescents between 10 to 16 years.^10^

However, two problems noted among the existing condition-specific questionnaires were: lack of a conceptual model explaining the theory behind the scale, and none of them contained items related to socioeconomic status of individuals seeking orthodontic treatment. Orthodontic treatment is subsidized only in certain countries and the uptake is based on the normative criteria. Many studies have noted a disparity between the complexity of malocclusion and psychological impact.^1^ ^,^ ^14^ ^-^ ^16^ Hence an uptake for treatment solely based on normative criteria is erroneous. Socioeconomic status is an important factor in seeking orthodontic care across the globe, especially with the increase in socioeconomic divide and ever increasing immigrants.^15^ Since pain and discomfort is not common among orthodontic problems, none of the conceptual model fits for malocclusion.^1^ Wilson and Cleary’s is a more comprehensive model, considering characteristics of the individual and also characteristics of the environment.^17^ Based on the studies of Benson et al.^18^ and Vedovello et al.,^19^ we hypothesized that socioeconomic status is an important contextual determinant in OHRQoL of subjects seeking orthodontic treatment, and accordingly we used a modified Wilson and Cleary model for our study. A similar model was previously used by Benson et al.,^18^ for studying OHRQoL status of children in the UK. Environmental factors like socioeconomic status, personal characteristics like self-esteem, psychological well-being and non-medical factors contributing to OHRQoL were considered in this model. 

Adolescence is a period of change both physically and psychologically. This is the period when self-esteem and emotional well-being takes a definite shape and gets stabilized.^11^ Considering the complexity of psychological changes happening during adolescent period, a single psychometric scale to cover the entire adolescence period cannot be considered adequate. One scale for the early adolescence (10-14 years) and another one for late adolescence (15-19 years) is appropriate to resolve this problem. To our knowledge, no condition-specific tool has been developed and validated for older adolescents, though MIQ can be used for young adolescents. There are only few studies which aimed to develop and validate a scale on a population-based sample.^20^ However most of them use a hospital-based subjects or convenience sample. The true constructs of a QoL instrument is not unveiled in such studies, hence a population-based study was planned to develop a valid questionnaire for older adolescents.

Thus, the aim of this study was to develop a psychometric scale, with appropriate socioeconomic considerations and minimum number of items, to explain the required constructs for a malocclusion-related quality of life instrument among older adolescents.

## MATERIAL AND METHODS

Ethics committee approval was obtained prior to study (M/02/2011/DCK). Informed consents were obtained from all the participants and their parents at all stages of study. By analyzing the items of validated tools and using the following qualitative steps, a draft item pool was developed.^4^ ^,^ ^5^ ^,^ ^7^ ^-^ ^9^ ^,^ ^12^

### Item generation

» Expert opinion: Ten orthodontists of varying experience levels were consulted and interviewed separately. Items in the existing tools with possible limitations in current sociocultural background were discussed and suggestions were recorded. 

» Key informant interview:15 subjects aged between 15 and 18 years with malocclusions, reported for treatment in orthodontics out-patient department, were interviewed by the principal investigator and opinions were recorded. Questions were asked about the affliction due to malocclusion in the proposed domains, based on a bio-psycho-social conceptual model.^17^ ^,^ ^18^ After interviewing each participant, their parents (father, mother or both, according to the availability) were interviewed separately, to register their views.

» Focus group discussions (FGD): Two sessions of FGD’s were conducted, in selected urban and rural schools. Each session involved one class of selected 20 students (10 males and 10 females) studying in plus-one grade, aged between 15 and 18 years. One moderator led the discussions with prepared topic guide and two recording clerks recorded the proceedings in audio. Semi-structured questions of neutral nature were asked, and care was taken to avoid leading questions. The sessions were concluded when no additional information was obtained. With the above qualitative methods, a draft item pool of 41 questions was developed. Two repeated questions were eliminated, reducing the number to 39. No attempt to categorize the items into subdomains was done at this stage. The response options being bipolar and similar for all items, were to be reversed for negatively worded items of the scale, for total score calculation.

### Item reduction

» Expert paneling: The draft items were presented to 10 experts, for ensuring content validity. The experts included 7 orthodontists, 2 pedodontists and 1 clinical psychologist. Items with the proposed five-point Likert response scale (1 = never, 2 = occasionally, 3 = little, 4 = yes, 5 = definitely yes) were presented to each expert separately. They were asked to grade it as “most relevant”, “relevant”, “can be avoided”, and “not relevant”. The responses were dichotomized, and a content validity Index (CVI) was estimated.^21^ Items with CVI of 0.8 and above were qualified for inclusion.^22^ Ranking of items based on relevance and arrangement in proposed domains were done at this stage by the experts. These draft items were presented to a convenience sample of 20 patients reported for treatment in orthodontics out-patient department, to ensure face validity.

### Translation and back-translation

The item pool developed in English was translated to Malayalam by 3 experts who are fluent in both languages. A best version was derived after comparing all three translations, by consensus. This Malayalam version was back-translated to English by another set of 3 experts fluent in both languages, and best matching translation was accepted as the draft Malayalam tool. One member among both panels was a linguistic expert. 

### Pretesting of the scale

The translated draft tool was pretested in a class of 35 students with prior permission from the school principal and class teacher. After dictating each item, lack of clarity, ambiguity, and level of understanding were identified. Items with these problems were reworded and submitted for expert review and later pretested in a different group of 15 subjects.

### Reliability of the new scale

The translated and pretested draft tool was piloted in a representative sample of 80 subjects. The questionnaire was administered in a specially arranged class. After 15 days, a repeat measure of the tool to the same group was performed. Data sheets of subjects who filled on both occasions (68 subjects) were subjected to analysis. Intraclass correlation coefficient (ICC) was calculated to ensure test-retest reliability.^23^ ^,^ ^24^

### Validation of the new scale

The draft tool was further tested for reliability and construct validation. The sample size required was 330 for this phase of the study (number of items x 10).^25^ ^,^ ^26^ However 450 subjects were recruited, among which 431 were included for data analysis. Those with severe dentofacial deformity due to syndromes, fluorosis or missing/fractured anterior teeth, cleft lip/palate and those with history or currently undergoing orthodontic treatment were excluded. Only those with signed informed consent and in the age range between 15 and 18 years were allowed to participate.

Four schools (two urban and two rural) were randomly selected from the higher secondary school list. The main investigator, with more than 15 years of experience in orthodontics, recorded normative malocclusion features and treatment need, while a second investigator administered the questionnaire. Intraexaminer reliability of the principal investigator was established prior to the study by repeating the recordings of Indices (IOTN-DHC and DAI) twice with a 14-day interval in 20 patients of the same age group. An ICC of 0.91 to 0.96 obtained for IOTN-DHC and 0.82 to 0.93 for DAI was considered good.

All examinations were performed under natural light, using a sterile mouth mirror and CPITN periodontal probe. To avoid the examiner fatigue, only 25 to 30 examinations were performed per day. One global question was included in the questionnaire for ensuring convergent validation. The normative need for orthodontic treatment was assessed using the Index of Orthodontic Treatment Need (IOTN)^3^ and the Dental Aesthetic Index (DAI).^2^ The socioeconomic status of the subjects was assessed based on the government recognized method of APL (Above Poverty Line) and BPL (Below Poverty Line) criteria.

### Statistical analysis

Statistical analysis was performed using SPSS (v. 16.0, SPSS Inc., Chicago, IL, USA). Score reversal of items worded in opposite manner was performed prior to data entry. A positive or negative item endorsement of more than 90% in one direction was considered to have little discriminatory power and decided to be excluded. 

Reliability of the scale was determined using Cronbach’s alpha. An alpha of 0.70 or above is essential to consider it as reliable.^26,27^ Item total correlation was estimated by correlating each item with the sum of the remaining items in the same domain, and a coefficient of 0.40 or above is required to retain that item. In addition, if an item was deleted, alpha was also calculated. If the reliability of the scale increased when an item was deleted, that item was not reliable for inclusion in the scale.

Principal component analysis with Kaiser normalization was done for factor extraction, and varimax rotation was performed for a rotated factor loading matrix. Loading of 0.40 or more was considered to be adequate.

Domain-wise and scale total mean scores were compared with normative malocclusion measures of IOTN-DHC and DAI, using independent *t*test, to ensure construct validation. Pearson correlations of the new scale with global question was done to ensure convergent validation. Validation hypothesis were tested at a significance level of *p*< 0.05. Kaiser-Meyer-Olkin sampling adequacy test ensured that the sample size was adequate for factor analysis. Factor ability of the data was tested using Bartlett’s test of sphericity, at a significance level of *p*< 0.01. Communality of the items was estimated and values above 0.60 were retained. Cut-off points for the new scale was derived using a quartile split procedure.

## RESULTS

The initial qualitative steps of tool development were done according to strict criteria, to ensure good content and face validity. Experts were of the opinion to include both positive and negative aspects of dental occlusion in the questionnaire. Five modified items of dental self-confidence scale proposed by Klages^9^ were selected for this purpose. However, 9 out of 10 panelists opined to change the name of the subscale to “Orthodontic self-confidence”. Inclusion of questions that reflect the socioeconomic background was unanimously agreed by all the experts and key informants. Seven new questions pertaining to socioeconomic background emerged out of the qualitative steps, but only two items could be retained in the final scale after factor analysis. Questions related to aesthetic aspects of the occlusion were included in the psychological domain. The majority of panelists opined that aesthetic aspect of dentition is reflected in the psychology of an individual. This was later confirmed by factor analysis. 

Out of the 39 items, 4 demonstrated a CVI below 0.80 after expert paneling. This reduced the item pool to 35. Pre-testing of the draft tool ensured its readability, level of understanding and lack of ambiguity among the responders. A Flesch-Kincaid Grade Level reading score of 8.1 indicated that the questionnaire was acceptable for an eighth grade student to read and understand.

Two items with ICC less than 0.75 were eliminated due to poor test-retest reliability. An initial Cronbach’s alpha of 0.883 was found to be good. Inter item and item total correlation ensured good internal consistency reliability. Domain-wise and scale total Cronbach’s α was found to be good (Table 1).

**Table 1:** Factor loading of the items of MRQoLQ subscales after Principal component analysis with varimax rotation, amount and percentage of variance explained by each factor (initial and rotated solution), α when a item was deleted from the whole scale and from the subscales, and α of each domains.

Item briefing	Psychological and socioeconomic	Orthodontic self confidence	Social interaction domain	Functional Impact	α - if item deleted from subscales	α - if item deleted from the scale
Unsatisfactory teeth position	0.684				0.854	0.913
Inferiority feeling	0.613				0.853	0.913
Concerned about opposite sex’s thinking	0.597				0.853	0.913
Friends made fun	0.698				0.863	0.915
Felt the need for brace treatment	0.659				0.854	0.913
Distressed seeing nice teeth of others	0.443				0.864	0.914
Opportunity to meet a doctor	0.670				0.857	0.915
Family income	0.577				0.866	0.915
Proud of my teeth		0.732			0.813	0.914
Satisfied with teeth position		0.710			0.811	0.913
Satisfied during smile		0.715			0.820	0.915
Satisfied when I see in mirror		0.690			0.813	0.912
Attractive to others		0.723			0.857	0.918
Kept away from public functions			0.607		0.889	0.916
Covers while speaking			0.848		0.653	0.915
Covers while laughing			0.821		0.655	0.915
Pronouncing certain words				0.601	0.706	0.918
Chewing problem				0.828	0.609	0.918
Avoid some food				0.810	0.629	0.918
Problems with jaw joints				0.493	0.716	0.918
Amount of variance (initial solution)	8.021	1.891	1.250	1.153		
Percentage of variance explained (initial solution)	40.104	9.454	6.250	5.765		
Percentage of variance explained (rotated solution)	19.936	17.399	12.445	11.793		
Cronbach’s α for each domain	0.874	0.854	0.825	0.728		

**Figure 1:**
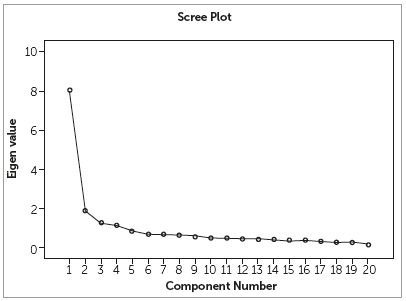
Scree plot showing component extraction based on Eigenvalue.

Principal component analysis based on Kaiser-Guttman criteria with Eigenvalue greater than one extracted four factors (Fig 1). Four components together explained 61.573% of the total variance. Orthogonal rotation using varimax derived a factor structure with optimized factor loading to derive a tool with minimum number of items. Few items with cross-loading and one item with no loading were eliminated, to get an improved solution. A final decision to retain 20 items was made, without compromising the variance. Percentage variance for each factor after rotation was as follows: first factor, 19.93; second factor, 17.39; third factor, 12.44 and fourth factor, 11.79.

Out of the four factors extracted, first factor explained 40.10% of the total variance and the items were of psychological nature. However the two new socioeconomic items were loaded in the first factor, contributing to the total variance, hence the new items were grouped as a subscale of psychological domain. Second factor extracted were items of orthodontic self-confidence scale (9.45%), third factor was social impact (6.25%) and fourth factor contained items of functional impairment (5.76%). The new scale had 20 items arranged in four domains: psychological (6 items) with a subdomain (2 socioeconomic items); orthodontic self-confidence (5 items), social impact (3 items) and functional impairment (4 items).

431 participants took part in the validation study, 41.5% of urban and 58.5% of rural background. 57.3% were from government schools and 42.7% were from private schools. Economic status was almost evenly distributed, 50.1% belonging to Below Poverty Line (BPL) and 49.9% Above Poverty line (APL). 37.6% of the subjects were males and 62.4% were females.

The difference in mean score of dichotomized IOTN-DHC and DAI, when compared with the mean score of newly developed Malocclusion-Related Quality of Life Questionnaire (MRQoLQ), was statistically significant (*p*< 0.001, Table 2). A positive correlation between Global Question and MRQoLQ score (r = 0.539, *p*< 0.01) ensured convergent validation. Independent *t* test comparing the mean scores of self-rated and interviewer-rated IOTN-AC also showed statistically significant difference with mean MRQoLQ score (*p*< 0.05).

**Table 2:** Validation of Malocclusion-Related Quality of Life Questionnaire (MRQoLQ) with dichotomized clinical normative criteria.

Normative variables	Category	MRQoLQ Mean and SD	t- value	Significance
IOTN - DHC	No need	45.34 ± 12.9	-3.975	p < 0.001*
	Definite need	53.02 ± 12.34		
DAI	No need	45.17 ± 12.80	-4.88	p < 0.001*
	Definite need	54.69 ± 12.02		
IOTN - AC (subject)	No need	45.5 ± 13.11	-2.896	p < 0.05**
	Definite need	50.86 ± 11.77		
IOTN - AC (investigator)	No need	45.73 ± 12.96	-3.14	p < 0.05**
	Definite need	53.81 ± 12.29		

The minimum possible score for the scale is 20 and maximum is 100. Cut-off points identified were: a score of 36 or less is good QoL; 37-55 is moderate QoL; and 56 or above is poor QoL. Maximum score obtained was 88, demonstrating good ceiling effect for the scale.

## DISCUSSION

Malocclusion is a public health concern in any country. In a fast-developing country like India socioeconomic barrier is still a hindrance to assess orthodontic care. In India orthodontic treatment is provided mainly in private clinics where the treatment charge ranges from few thousands to lakhs of Indian rupees. All the available condition-specific tools to assess malocclusion-related quality of life has been created for English-speaking developed countries.^7^ ^-^ ^10^ Translation and cross-cultural adaptation is done prior to its application in countries of different language and culture. No condition-specific malocclusion scale has currently incorporated socioeconomic status of the subjects, even though it has influence in malocclusion-related quality of life among adolescents.^18^ ^,^ ^19^ QoL is an abstract concept and is not easily definable and cannot be fragmented. Studies have shown a definite impact of malocclusion on QoL, but to varying extent.^1^ ^,^ ^13^ ^,^ ^28^

The present study aimed to develop a new condition-specific psychometric tool to assess malocclusion-related quality of life in adolescents, with a minimum number of items and maximum variance. The criteria of Guyatt and Juniper was followed in the scale development process. Initial item pool was derived by a combination of certain qualitative steps and modified items from exiting tools. The use of items from existing tools is recommended as it has already passed its validity testing.^23^ Body image concerns among adolescents are strong, having influence in psychological and social adjustments, and educational success.^11^ Hence, age group selected was important and not subjected to previous study for developing a condition-specific measure.

Seven new items pertaining to socioeconomic status were included in the item pool, and two could be retained after factor analysis. The recommended minimum number of items for a domain is two.^27^ However they were allocated in the psychological domain and hence considered as a subscale of it. 

Principal component analysis extracted four factors. The first extracted factor had 6 questions pertaining to the psychological aspects of malocclusion. According to many investigators, psychological impact is still the main domain affected by malocclusion.^29^ Socioeconomic factors are also of concern where public aid is not available for orthodontic treatment due to cosmetic nature of the problem. The two socioeconomic items were allocated in the psychological domain, indicating that economic status for accessibility to orthodontic care psychologically affects patient. Esthetic aspects of the occlusion were similarly considered indistinguishable from psychological impact by experts and hence considered as part of it.

The social impact and functional impairment due to malocclusion were clearly distinguishable and allocated separately, hence considered as distinct domains. The need of assessing positive aspects of oral health is important in a QoL scale.^8^ ^,^ ^9^ This made us to include 5 modified items of Dental Self Confidence scale of Klages et al.^9^ These items are related to dental alignment and smile *per se*, and not related to integrity of teeth; thus the domain was named as Orthodontic Self-confidence (OSC). The response options of this scale were in reverse order, hence score reversal was done during analysis stage. 

Any psychometric scale should be reliable and valid.^23^ Reliability refers to repeatability of the scale, and validity refers to measuring what a scale is intended for. Cronbach’s α of 0.88 was found to be good. Domain-wise alpha was also good, except for the functional impact domain (0.728). An alpha of 0.7 or above is considered good for a new scale,^23^ and for more established ones it should be above 0.8. ICC of 0.80 ensured good test-retest reliability. Validity of the scale was ensured by factor analysis and hypothesis testing with normative parameters. Convergent validation using Global Question and self-rated IOTN-AC was found to be good. Construct validation using IOTN-DHC and DAI was found to be statistically significant (*p*< 0.05), ensuring the ability of the scale to distinguish those with treatment need and those without need.

Limitations of the study include few subjects with grade IV and grade V of IOTN-DHC (11.6%), due to the cluster sampling strategy. The influence of buffers like self-esteem, psychological well-being,^30^ and social support mechanism dilutes true estimation of OHRQoL. Presence of other dental problems like dental caries and periodontal problems are potential confounders in the estimation of OHRQoL. Hence further validity and responsiveness testing of the scale is necessary. 

## CONCLUSIONS

This new scale named Malocclusion-Related Quality of Life Questionnaire (MRQoLQ) contains 20 items arranged in four domains: psychological, with a socioeconomic subdomain; orthodontic self-confidence; social impact and functional impairment. Initial study has shown that the scale is reliable and valid for the tested Indian population.

Socioeconomic factors of patients seeking orthodontic treatment cannot be neglected in the light of this study. Also, uptake of patients for subsidized treatment based on mere normative criteria is not enough; instead, the use of a psychometric tool reflecting socioeconomic domain is recommended.
